# Editorial: Plant Genome-Epigenome Integrity Under Environmental Stress

**DOI:** 10.3389/fpls.2020.584126

**Published:** 2020-08-28

**Authors:** Paula Casati, Teresa Roldán-Arjona, Jean Molinier

**Affiliations:** ^1^Centro de Estudios Fotosinteticos y Bioquimicos (CEFOBI-CONICET), Universidad Nacional de Rosario, Rosario, Argentina; ^2^Departamento de Genética, Universidad de Córdoba, Córdoba, Spain; ^3^Institut de biologie moléculaire des plantes (UPR2357-CNRS), Strasbourg, France

**Keywords:** chromatin dynamics, transposable elements, plant epigenome integrity, environmental stress, DNA damage and repair

Plants, due to their sessile life style, need to cope with different environmental stresses to allow proper growth and development. DNA, in association with histones, is packaged into chromatin. Several changes in chromatin structure, such as post-translational modifications of histones and DNA methylation strongly affect chromatin accessibility. In past years, it has been demonstrated that biotic and abiotic stresses trigger changes in chromatin structure, allowing modification of transcriptional programs and modulation of genome structure. Chromatin compaction and its accessibility to various factors, such as transcription factors or DNA repair enzymes, regulate transcriptional activity and also DNA repair efficiency. Chromatin remodelers, histone readers/modifiers and DNA methylases/demethylases are among the predominant factors that contribute to genome and epigenome dynamics.

Upon environmental stress exposure and induction of the appropriate molecular responses, genome and epigenome organization must be efficiently restored. In other words, chromatin accessibility/architecture has to be reestablished allowing accuracy in the transcriptional regulation and also in genome 3D organization. Moreover, most of the DNA repair pathways used to repair environmentally-induced DNA damage are DNA synthesis-dependent repair processes. Thus, in addition to the need of accuracy during DNA synthesis to maintain genetic information integrity, the epigenomic landscape should also be efficiently and accurately restored. Altogether, different studies are providing accumulating evidence that pathways involved in the maintenance of genome and epigenome integrity have to be tightly coordinated.

In line with these observations, this Research Topic focuses on genome and epigenome surveillance processes that are mobilized upon environmental stress exposure. In this issue, three review articles summarize various aspects of how chromatin accessibility regulates plant responses under different environmental stress conditions. In Bourbousse et al., the authors describe present knowledge on how light can modulate the composition and the spatial distribution of chromatin domains. Different light signaling pathways that require chromatin-based mechanisms and play critical roles in plant phenotypic plasticity are described. Some changes in chromatin structure after light exposure regulate transcription in a locus-specific manner but, interestingly, light-dependent signals can also modulate higher-order chromatin organization. This review also discusses about the links between light signaling and the epigenome and how different chromatin regulatory layers may interconnect during plant adaptive responses to light.

The review by Hu et al. focuses on a particular post-translational histone modification, histone lysine acetylation. Recent data has shown that histone acetylation is essential for epigenetic regulation during plant responses to stress. Histone acetylation requires the action of enzymes that uses primary metabolites as substrates or cofactors; these compound levels are also affected by stress and growth conditions. Strikingly, results have shown that besides acetylation, histone lysine residues can alternatively be acylated by similar enzymes than those participating in the deposition and removal of histone acetylation. This review summarizes results on the relationship between histone acetylation and acylation under stress conditions in plants and provides a model showing the interplay between primary metabolism and epigenetic regulation of genes to adapt to stress conditions.

On the other hand, Durut and Mittelsten Scheid discuss the role of noncoding RNAs (ncRNAs) in double-strand break (DSB) repair. Genome stability is constantly threatened not only by environmental factors but also by endogenous processes that damage DNA. If DNA lesions are not correctly and timely repaired, then these lesions can lead to mutations and genomic rearrangements. Recently, ncRNAs have been reported to participate in the maintenance of genome stability. This review describes how these ncRNAs participate in the repair of DSBs. The authors also discuss how genome editing approaches, in particular CRISPR/Cas systems, can be used in DNA repair studies in plants.

In addition, two research papers describe novel aspects of the role of chromatin proteins under stress conditions. In the Vyse et al. paper, the role of chromatin regulators under low temperature conditions was investigated. The authors show that under non-stress conditions, genes that participate in cold responses are associated with the repressive chromatin mark histone H3 lysine 27 trimethylation (H3K27me3). While target genes are quickly activated by cold, the association of H3K27me3 with these genes is not always removed. However, a large group of chromatin genes are temperature regulated, such as different DNA and histone demethylase genes and particular histone variants. The analysis of previous published transcriptomic datasets shows that these chromatin remodeling genes are also regulated by alternative splicing under cold conditions. This work provides a profiling platform for the analysis of chromatin regulatory genes and their regulation in response to cold stress.

The role of the histone lysine methyltransferase SET DOMAIN GROUP 8 (SDG8), which trimethylates histone H3 in lysine 36 in Arabidopsis defense responses is presented in Zhang et al. *sdg8* mutants are not only deficient in H3K36 methylation, but they also display a higher sensitivity to the bacterial pathogen *Pseudomonas syringae*. In these mutants, the accumulation of salicylic acid is significantly higher in healthy plants, but after infection, levels decreased very quickly compared to WT plants. Moreover, expression levels of defense-related genes were also altered in *sdg8* plants. Interestingly, while global levels of histone methylation were not affected in WT plants, increases in H3K4 and H3K36 methylation and RNA POLYMERASE II (RNAPII) loading were found associated with some defense genes after treatment with salicylic acid. In contrast, in *sdg8* mutants, the increase in H3K36me3 associated with defense genes was not observed, suggesting that SDG8 is important for H3K36 methylation during immune responses. Finally, this work demonstrates that SDG8 physically interacts with the RNAPII, which provides a possible link between H3K36me3 deposition and RNAPII loading during plant defense responses to bacterial pathogens.

Finally, Tomkova and Berr revise a new version of the book: Epigenetics in Plants of Agronomic Importance: Fundamentals and Applications. This book analyses and summarizes recent data on the role of epigenetic mechanisms modulating plant development and stress responses. Also, the authors discuss the importance of understanding how epigenetic modifications regulate plant development under stress conditions as potential tool for sustainable agriculture under climate change conditions.

To conclude, this Research Topic issue highlights the importance of understanding how changes in chromatin structure modulate plant responses in a changing environment.

## Author Contributions

PC wrote the first draft of the article. TR-A and JM made a substantial, intellectual contribution to the work and approved it for publication.

## Conflict of Interest

The authors declare that the research was conducted in the absence of any commercial or financial relationships that could be construed as a potential conflict of interest.

